# Effect of different root canal irrigants on push-out bond strength of two novel root-end filling materials

**DOI:** 10.1186/s12903-023-02858-7

**Published:** 2023-04-02

**Authors:** Nada Omar, Rasha M. Abdelraouf, Tamer M. Hamdy

**Affiliations:** 1 Restorative and Dental Materials Department, Oral and Dental Research Institute, National Research Centre (NRC), Giza, Dokki 12622 Egypt; 2 Biomaterials Department, Faculty of Dentistry, Cairo University, Cairo, 11553 Egypt

**Keywords:** Bond strength, Push-out, Root-end filling, Irrigants, Chlorhexidine, Sodium hypochlorite, MTA, PMMA, Hydroxyapatite, Open apex, Apical seal

## Abstract

**Background:**

The aim of this in vitro study was to evaluate push-out bond strength of different root-end filling materials using various irrigant solutions.

**Methods:**

A push-out bond strength test was performed to evaluate the bond strength of two experimental root-end filling materials: namely, nano-hybrid mineral trioxide aggregate (MTA) and polymethyl methacrylate (PMMA) cement filled with 20% weight nano hydroxyapatite (nHA) fillers compared to conventional MTA. The irrigant solutions employed were sodium hypochlorite (NaOCl) in concentrations 1%, 2.5% and 5.25% and 2% chlorhexidine gluconate (CHX) followed by application of 17% ethylene diamine tetra-acetic acid (EDTA). A freshly extracted sixty single-rooted human maxillary central incisors were used. The crowns were removed, the canal apex was widened to simulate immature teeth. Each type of irrigation protocols was performed. After application and setting of the root-end filling materials, a slice of one mm thickness was cut transversely from the apical end of each root. Specimens were stored for 1 month in artificial saliva and were subjected to a push-out test to evaluate the shear bond strength. Data were analyzed using two-way ANOVA and Tukey test.

**Results:**

The experimental nano-hybrid MTA showed the highest significant push-out bond strength values when irrigated by NaOCl at several concentrations (1%, 2.5% and 5.25%) (P< 0.05). Meanwhile, irrigation with 2% CHX resulted in highest bond strength values in nano-hybrid white MTA (18 MPa) and PMMA filled with 20% weight nHA (17.4 MPa) with nonsignificant difference between them (p = 0.25). In each root-end filling material, irrigation with 2% CHX led to the highest significant bond strength, followed by NaOCl 1%, while the least significant bond strength was produced after irrigation with NaOCl 2.5% and 5.25% (P< 0.05).

**Conclusion:**

Considering the limitations of this study, it may be concluded that the application of 2% CXH and 17% EDTA provides superior push-out bond strength to root canal dentin compared with irrigation with NaOCl irrigants and 17% EDTA, experimental nano-hybrid MTA root-end filling material provides enhanced shear bond strength than conventional micron-sized MTA root-end filling material.

## Background

Success of endodontic procedures depends mainly on a hermetic apical seal between the root canals and surrounding tissues [1]. The main purpose of the root-end filling materials is to enhance the apical sealing to avoid leakage of irritants and to prevent the reentering of microorganisms [2–5]. Therefore, a proper chemo-mechanical preparation of the root canal is essential to eliminate bacteria and their byproducts, in addition to removal of the inflamed and infected tissue [6, 7]. It is necessary to combine mechanical instrumentation with chemical irrigants to ensure the removal of smear layer to enhance the adaptability of root canal filling materials [7, 8].

The most widely used root canal irrigant solutions are NaOCl, CHX and EDTA. Their main function is to eliminate bacteria, bacterial byproducts, and smear layer, leaving a clean surface of dentin [9]. NaOCl is considered a proteolytic agent with a potent tissue-dissolving action, but its dissolving capability relies on its concentration and duration of application [10, 11]. NaOCl is primarily utilized as an endodontic irrigant solution due to its ability to dissolve organic tissues and its antibacterial effect [12]. Aggressive application of NaOCl could resulted in dramatic reduction in the bond strength mainly due to formation of an oxygen-rich layer on the dentinal surface, which occurs by cleavage of NaOCl into chlorine and oxygen [13].

Currently, CHX has been introduced in root canal therapy to disinfect the canal. Chlorhexidine possesses antimicrobial action and is considered a broad-spectrum antiseptic solution with a limited tissue-dissolving ability [14, 15]. EDTA is a potent decalcifying agent that eliminates the inorganic component of the smear layer [16]. It could be used as 17% EDTA in combination with other root canal irrigant solutions to ensure clearance of the smear layer and to gain a synergistic effect to enhance antimicrobial potentiality [9, 17, 18]. Additionally, it serves as an antioxidant agent that helps in the removal of the oxygen-rich layer, subsequently enhancing the interaction between the dentin and root-end filling materials [19]. Proper endodontic treatment requires a continuous improvement in endodontic filling materials in conjunction with enhancement of newly designed root canal instruments and instrumentational techniques [20–22].

MTA is the most prominent root-end filling material due to its adequate sealing ability, biocompatibility and bioactivity. Besides these advantages, MTA has some drawbacks that interfere with adequate obturation, such as poor handling characteristics and prolonged setting time, in addition to possibilities of discoloration of the remaining tooth structure [23, 24].

Nanotechnology has been employed in several branches of dentistry due to the great potential of nanomaterials to improve mechanical properties, inhibit biofilm development, eradicate microorganisms, promote remineralization of the tooth structure by reducing demineralization, eliminate dentin hypersensitivity and enhance tissue regeneration [25–28].

Nano-hybrid MTA is a newly introduced material that contains a mixture of nanoparticles of silica oxide, alumina oxide, titanium oxide and nHA, in addition to micro-sized silica, for more favorable physicochemical and handling properties to obtain maximum clinical and biological merits [26, 29]. Moreover, calcium-phosphate based materials have been extensively used to promote remineralization and repair of dental hard tissues due to their bioactivity and their potential to liberate calcium and phosphorus ions [27, 30–33]. Moreover, nHA exhibits both biocompatible and bioactive properties, with enhanced physical, mechanical and biological properties [28, 34–36]. *Daokar et al.*. in 2021, concluded that the addition of nHA fillers to conventional glass ionomer cement increased bond strength to dentin mainly due to the smaller particle size of nHA, which enhances the infiltration capacity to tooth surface, thereby reinforcing the bonding strength [37]. Polymeric material is of a great interest in dentistry due to its improved mechanical and thermal properties, in addition to its sufficient flowability, especially when utilized in low filler concentration [38–43]. The bond quality and integrity of restorations are strongly related to their bond strength [44]. Bond strength among dentin and the root-end filling material is of extreme significance in the clinical success of root canal treatment [45, 46].

The bond strength is commonly assessed by the push-out bond strength test [45, 47]. The push-out test has been considered the most valid evaluation of the bond strength of a material to its surrounding dentin [48, 49]. In cases of a wide-open apices, it is difficult to achieve an adequate condensation of MTA as the material could be extruded outside the apex [46]. Therefore, there is a great demand for development of novel root-end filling materials that promote apical seal.

This *in-vitro* study was conducted to assess the effect of using a variuos irrigants which are: NaOCl 1%; NaOCl 2.5%; and NaOCl 5.25% (full concentration) and 2% CHX respectively, followed by application of 17% EDTA on two experimental root-end filling materials namely; nano-sized MTA and PMMA cement filled with nHA fillers using push-out test compared to traditional MTA on open apex root canal.

The null hypothesis was that there was no significant difference regarding push-out bond strength when using NaOCl (1%, 2.5% and 5.25%) and 2% CHX followed by 17% EDTA among the following three root-end filling materials: conventional MTA (Angelus), experimental Nano-hybrid MTA (Nano white MTA), and experimental PMMA filled with 20% weight nHA.

## Methods

### Root-end filling materials

Two experimental root-end filling materials were used: namely, Nano-hybrid MTA (*NanoTech Egypt labs, Egypt*) and PMMA cement (*Cemex®Isoplastic, Tecres, Italy*) incorporated with 20 weight% nHA fillers (*NanoTech Egypt labs, Egypt*) manually as described previously [28], and compared to conventional MTA-based material (*Angelus, Londrina, PR, Brazil*) as shown in (Table 1).

**Table 1:** The composition of the root-end filling materials used in this study

Materials	Manufacturers	Composition
Nano-hybrid MTA (Nano white MTA)	*NanoTech Egypt labs, Egypt*	Nano-silica, nano-alumina, nano-titanium oxide, nanohydroxyapatite, tri-calcium aluminate, calcium sulfate, zeolite and strontium doped calcium polyphosphate.
Cemex ® Isoplastic	*Tecres, Verona, Italy*	Powder: PMMA, styrene, benzoyl peroxide and barium sulphate Liquid: MMA, N, N-Dimethyl-p-toluidine and hydroquinone
Nano-sized HA	*NanoTech Egypt labs, Egypt*	Nano-hydroxyapatite
Angelus MTA	*Angelus, Londrina, PR, Brazil*	Tri-calcium silicate, di-calcium silicate, tri-calcium aluminate, ferroaluminate tri-calcium, calcium oxide, bismuth oxide

### Experimental groups

The samples were randomly divided into three main groups (n = 20/group), according to the root-end filling materials used: Conventional MTA (Angelus); Nano-hybrid MTA; PMMA cement filled with nHA. In each group, samples were further sub-divided into four sub-groups (n = 5) according to the root canal irrigants used: 1% NaOCl, 2.5% NaOCl, 5.25% NaOCl (full concentration), and 2% CHX.

### Specimen size determination

Sample size was calculated using sample size calculator software program (G. power 3.19.2) based on research published by *Fisher et al.*, *Ahmed* et al., and *Akbulut* et al. [50–52]. Sample size calculation was based on 95% confidence interval and power of 90% with α error of 5%.

### Specimen preparation

All experiments were performed in accordance with relevant guidelines and regulations by the Research Ethics Committee of the Faculty of Dentistry, Cairo University (Approval No: 24/9/22). A freshly extracted set of sixty single-rooted human maxillary central incisors with straight and single canals were collected, visually inspected and radiographically examined. The exclusion criteria included carious, cracked, previous root canal treatment, and fractured teeth. Teeth were cleaned carefully using curettes to eliminate any remnants of the soft tissues and calculus.

Specimens were prepared following a previous protocol reported in the literature [53, 54]. The crowns were removed 1 mm below the cementum-enamel junction using a water-cooled low-speed diamond drill (NTI, Superflex, Kerr Dental Co., California, USA). The root canals were initially widened using K-20 files (Dentsply/Maillefer, Ballaigues, Switzerland). Next, enlargement was proceeded using ProTaper rotary instruments (PTU; Dentsply Maillefer, Ballaigues, Switzerland) in a standardized sequence up to no. 40 (F4). The canals were over instrumented using Peeso-reamers (size 1–6) (Mani Inc., Tochigi, Japan) up to size 6 (1.7 mm), which passed 1 mm beyond the canal apex to simulate immature teeth [55].

### Irrigation protocols

During the root canal preparation, the root canals were initially irrigated with 1 mL of one of the previously mentioned irrigants for 1 min after each instrumentation using a side-vented needle (24/0.4), and then with 5 mL sterile distilled water followed by 1 mL 17% EDTA (Maquira, Maringa, Brazil) as the final rinse after the whole instrumentation procedure. Lastly, root canals were flushed with 5 mL sterile distilled water and then dried using sterile paper points [17, 56].

### Application of root-end filling materials

Each root-end filling material was mixed according to the manufacturer’s instructions and condensed using a micro plugger in the previously prepared root canals. After the filling process was performed, a cotton pellet was used to clean the surface of the roots.

### Sectioning of apical third of the roots and storage

After setting of the filling materials, a slice of one mm thickness was cut transversely from the apical end of each root (perpendicular to the roots’ long axis) using a diamond-coated saw (Isomet, Buehler Ltd., USA) under water coolant.

The slices were then immersed in artificial saliva (Laboratory of Biochemistry Faculty of Pharmacy, Cairo University, Cairo, Egypt) (pH 7.4) and stored in an incubator (CBM, S.r.l. Medical Equipment, 2431/V, Cremona, Italy) at 37 ± 1° for 1 month, with the artificial saliva changed every three days. After 1 month, the specimens were subjected to a push-out test to evaluate the shear bond strength.

### Push-out test

The root-end filling material in each slice was exposed to compressive loading using a universal testing machine (Model 3345; Instron Industrial Products, Norwood, MA, USA) with a crosshead speed of 1 mm/min. A steel plunger (1 mm diameter) was used to apply compressive load on only the filling material without contacting the root. The diameter of each root-end filling material was then measured, and the apical and coronal radius was calculated using a digital caliber (Pachymeter, MEDA CO., LTD, Tianjin, China). Each root slice was mounted in a metallic custom-made fixture with circular middle hole.

The specimens were placed with the apical aspect facing the plunger tip to avoid stressing the surrounding dentin. Push-out bond strength values were recorded in Newton and converted into MPa. Bond strength was calculated as the force (N) of dislodgement divided by the surface area (A) of the bonded interface (mm^2^), using the following equation [57]:

$$[A = (\pi \, \times \,r1x\,\pi \, \times \,r2)\,L],$$

where

*π* is the constant 3.14

r1 apical radius, r2 coronal one,

$$L = {[{(r1 - r2)^2} + {h^2}]^{0.5}}$$

h is the thickness of the specimen in mm.

Bond failure was displayed by extrusion of root-end filling material and confirmed by sudden drop in load-deflection curve. The push-out bond strength was determined for each root disk.

### Statistical analysis

Statistical analysis was performed by utilizing Statistical Package for Social Sciences (SPSS) 16.0 statistical software (SPSS, IBM, Chicago, USA). Analysis of Variance (Two-way ANOVA) and Tukey test were employed to compare the mean push-out bond strength values (MPa) for the different root-end filling materials after using various irrigants. The significance level was set at P ≤ 0.05.

## Results

The mean bond strength values (MPa) for all groups are represented in (Table 2). The experimental nano-hybrid MTA (Nano white MTA) showed the highest significant push-out bond strength values when irrigated by NaOCl at several concentrations (1%, 2.5% and 5.25%) (P-value = 0.0001, 0.02, 0.02 respectively), when compared to conventional MTA (control) and experimental PMMA filled with 20% weight nHA.

No significant difference in bond strength was detected between conventional MTA (control) and experimental PMMA filled with 20% weight nHA when irrigated by NaOCl 2.5% and 5.25% (P-value = 0.15 and 0.94 respectively). While using NaOCl 1%, the conventional MTA showed higher bond strength (10.1 MPa) than experimental PMMA filled with 20% weight nHA (9 MPa) (P-value = 0.01).

Meanwhile, irrigation with 2% CHX resulted in the highest bond strength values in nano-hybrid white MTA (18 MPa) and PMMA filled with 20% weight nHA (17.4 MPa) with a nonsignificant difference between them (P-value = 0.25). Their values were significantly higher than conventional MTA (12 MPa) (P-value = 0.0001).

In each root-end filling material, irrigation with 2% CHX led to the highest significant bond strength. In conventional MTA (control), the 2% CHX had P-values = 0.02, 0.0001 and 0.0001 compared to NaOCl 1%, 2.5% and 5.25% respectively. While in both the experimental Nano-hybrid MTA (Nano white MTA) and the experimental PMMA filled with 20% weight nHA the P-value was 0.0001 between 2% CHX and other irrignats.

In each root-end filling material, irrigation by NaOCl 1% gave intermediate bond strength values which was lower than CHX but significantly higher than NaOCl 2.5% and 5.25%. In conventional MTA (control), the NaOCl 1% had P-values = 0.04 and 0.001 compared to NaOCl 2.5% and 5.25% respectively. While in the experimental Nano-hybrid MTA (Nano white MTA) the P-value was 0.0001 compared to both irrigants NaOCl 2.5% and 5.25%. Meanwhile, the experimental PMMA filled with 20% weight nHA this P-value was 0.004.

In each root-end filling material, the least significant bond strength was produced after irrigation with NaOCl 2.5% and 5.25% (with no significant difference between these two groups where P-value = 0.1).

**Table 2:** The mean push-out bond strength values (MPa) for the different root-end filling materials after using various irrigants

Root-end filling Irrigant solutions	Control (Gold standard) Conventional MTA (Angelus)	Experimental Nano-hybrid MTA (Nano white MTA)	Experimental PMMA filled with 20% weight nHA	P-value
1% NaOCl	10.1 ± 0.3 ^b^ II	15 ± 0.2 c II	9 ± 0.1 ^a^ II	P ≤ 0.01*
2.5% NaOCl	9 ± 0.3 ^a^ I	10 ± 0.2 ^b^ I	8.4 ± 0.1 ^a^ I	P = 0.02*
5.25% NaOCl	8 ± 0.2 ^a^ I	9.3 ± 0.3 ^b^ I	7.9 ± 0.3 ^a^ I	P = 0.02*
2% CHX	12 ± 0.3 ^a^ III	18 ± 0.2 ^b^ III	17.4 ± 0.1 ^b^ III	P = 0.0001*
P-value	P ≤ 0.04*	P = 0.0001*	P ≤ 0.004*	

Mean with different small letters in the same row indicate statistically significance difference, while mean with different capital roman letters in the same column indicate statistically significance difference *; significant (P < 0.05)

## Discussion

An effective endodontic treatment is based on the combination of adequate instrumentation, irrigation and root-end filling materials. This study provides important information regarding the effects of the most often used irrigant in daily clinical practice in relation to the newly introduced root-end filling materials. The correct selection of the necessary irrigant helps endodontists to promote a hermetical apical seal and achieve a sufficient bond strength to improves endodontic outcomes [58].

An ideal root-end filling material is required to be stable against the forces of dislodging and displacement. Push-out bond strength test is a practical and reliable method to assess the adaptability between the material and the surrounding dentin and to evaluate the dislodgement resistance of root-end filling materials to confirm their effectiveness [59, 60].

MTA is considered a promising root-end filling material that could guard against bacterial leakage, resist dislodging forces and promote a hermetical seal to the dentinal wall. Although MTA has some drawbacks, there is a great demand to evaluate novel root-end filling materials [23, 24].

Different irrigants were used in this study to evaluate their effect on push‑out bond strength of experimental nano-sized MTA and PMMA cement filled with nHA fillers on open apex root canal compared to traditional MTA.

NaOCl is the most commonly used irrigant due to its antimicrobial effect, lubricant activity and tissue‑dissolving capability [61]. CHX has been used during root canal treatment because of its antimicrobial effect and lower toxicity, in addition to its substantivity [62, 63] and ability to increase the surface energy of the dentin and to promote the adhesion between restorative material and dentin by inhibition of matrix metalloproteinases (MMPs) activity [64].

This study sought to evaluate the effect of different irrigant solutions (NaOCl (1%,2.5% and 5.25%) and 2% CHX) on the push-out test of MTA, nanosized MTA, and PMMA filled with nano HA materials to root dentin.

According to the above findings, the null hypothesis was rejected because the various kinds of irrigant solutions and their concentrations as well as the composition of the root-end filling materials had a significant difference on push-out bond strength. The highest mean push-out bond strength values of the nano-hybrid MTA (Nano white MTA) when irrigated by NaOCl at several concentrations (1%, 2.5% and 5.25%) when compared to the other groups may be attributed to their nanoparticles. The nanoparticle constituents led to a great surface area, which may have enhanced the rate of adhesion of the root-end filling materials to the tooth structure [29, 65, 66]. This finding was in accordance with study conducted by *Saghiri et al.* who concluded that Nano white MTA provided a higher bond strength value than conventional MTA [67].

Moreover, the presence of strontium doped calcium polyphosphate may be responsible for release of calcium, phosphorous and strontium ions, which could be deposited at the interface and obliterate the gaps between the dentin and root-end filling materials, consequently promoting bioactivity as well as increasing frictional resistance of the root-end filling materials and thereby bonding strength [60, 66, 68].

Furthermore, irrigation with NaOCl 2.5% and 5.25% using conventional MTA (control) and experimental PMMA filled with 20% weight nHA gives equal results, which may be due to the lower liberation of calcium and phosphorous ions and the lower surface energy of the macro-sized particles of conventional MTA than that of nano-hybrid MTA [29]. Furthermore, the increase in NaOCl concentration may adversely affect the bond strength, possibly due to the increased elimination of the organic matrix from the dentin, providing a little bonding surface [17, 69]. Moreover, PMMA filled with 20% weight nHA could be greatly affected by the action of chlorine due to the induced degradation effects on the polymeric matrix [70].

On the other hand, using the NaOCl 1% irrigant gives a higher bond strength when using conventional MTA than experimental PMMA filled with 20% weight nHA, which may be due to the lower adverse effect of the lower concentration of NaOCl on the bond strength of conventional MTA, while the deterioration effect of even lower than NaOCl 1% on the polymeric matrix of PMMA is critical [69, 71]. A previous research conducted by *Hu et al.* was reported that irrigation with 0.5% NaOCl, is recommended during root canal therapy to minimize any deterioration in bond strength [72].

Meanwhile, irrigation with chlorohexidine using either nano-hybrid white MTA or PMMA filled with 20% weight nHA does significantly increase the bond strength, which may be due to the fact that chlorohexidine yielded the highest bond strength between dentin surface and root-end filling materials. This finding may be attributed to the chemical properties of CHX, in addition to their ability to strongly bind to the root surface pellicle, hydroxyapatite and tooth [73]. Furthermore, the improvement in the bond strength of the root-end filling materials containing nano-sized particles may be due to their composition as the reduced size of nano-sized particles improves the penetration of root-end filling materials into dentinal tubules, hence increasing bond strength [74].

Finally, in each tested root-end filling material, the irrigation with 2% CHX led to the highest significant bond strength, followed by NaOCl 1%. The least results were demonstrated after irrigation with NaOCl 2.5% and 5.25%. This observation may be due to the detrimental effect of NaOCl irrigant on dentin bond strength, which may be due to the elimination of the organic matrix from the treated dentin, leaving a little bonding surface [69]. *Fibryanto et al.* concluded that root canal irrigation with NaOCl 2.5% and 5.25% caused collagen degeneration of the dentin, and the higher the concentration, the more pronounced was the reduction of the collagen content of the dentin, with a subsequent reduction in bond strength [10]. The same finding was observed by other study conducted by *Dinesh et al.* and *Ciapała et al.* They concluded that irrigation with 2% CHX provided a higher bond strength [75, 76]. Furthermore, the lack of adverse effects of the application of 2% CHX on the bond strength was reported in other studies [77–79]. While, other studies reported a decrease in bond strength upon application of 2% CHX irrigant [8, 80, 81]. Moreover, *Santos et al.* study was noted a significant reduction in bond strength values when using different concentration of NaOCl combined with 17% EDTA irrigant. However, irrigation with 2% CHX and 17% EDTA showed no adverse effects on bond strength [82].

Moreover, the irrigation employed with NaOCl using PMMA filled with 20% weight nHA may produce chloramine-derived radicals, which cause an early chain termination and hence in-complete polymerization [69]. Additionally, NaOCl acts as an oxidizing agent that breaks down into chlorine and oxygen and consequently conflicts with the free-radical polymerization of the PMMA and adhesion into the dentin [19, 83]. The results of the current study was in accordance with research performed by *Ari et al.*, who concluded that the irrigation with NaOCl reduce the bond strength of resin cements [84].

The current study has some limitations. First, only teeth with straight canals were involved in the study. Second, the difference in the amount of minerals and organic ingredients among the collected extracted teeth may affect the results. Third, the mode of failure was not assessed in such study. Fourth, the presence of many variables may be confusing the interpretation of the results.

## Conclusion

Within the limitations of the current in vitro study, the following can be concluded:

1. Experimental nano-hybrid MTA root-end filling material could be used as a substitute to the conventional micron-sized MTA root-end filling material regarding shear bond strength.
2. Combination of 2% CXH and 17% EDTA irrigant solutions are more effective regarding shear bond strength than that of NaOCl.
3. NaOCl in concentrations of 2.5% and 5.25% adversely affects the shear bond of the root-end filling material, especially when used with polymeric based material.

## Data Availability

The data that support the findings of this study are available from the corresponding author upon reasonable request.

## Abbreviations

NaOCl: Sodium hypochlorite
CHX: Chlorhexidine gluconate
EDTA: Ethylenediaminetetraacetic acid
MTA: Mineral trioxide aggregate
PMMA: Polymethyl methacrylate
nHA: Hydroxyapatite
MMPs: Matrix metalloproteinases.
